# Femtosecond Laser Fabrication of Monolithically Integrated Microfluidic Sensors in Glass

**DOI:** 10.3390/s141019402

**Published:** 2014-10-17

**Authors:** Fei He, Yang Liao, Jintian Lin, Jiangxin Song, Lingling Qiao, Ya Cheng, Koji Sugioka

**Affiliations:** 1 State Key Laboratory of High Field Laser Physics, Shanghai Institute of Optics and Fine Mechanics, Chinese Academy of Sciences, P.O. Box 800-211, Shanghai 201800, China; E-Mails: hef@siom.ac.cn (F.H.); superliao@vip.sina.com (Y.L.); jintianlin@siom.ac.cn (J.L.); sjx7013@siom.ac.cn (J.S.); llq198477@126.com (L.Q.); 2 RIKEN Center for Advanced Photonics, Hirosawa 2-1, Wako, Saitama 351-0198, Japan

**Keywords:** femtosecond laser, microfabrication, glass material, micro-/nanofluidics, lab-on-a-chip, optofluidics, electrofluidics, surface-enhanced Raman-scattering, lab-on-fiber

## Abstract

Femtosecond lasers have revolutionized the processing of materials, since their ultrashort pulse width and extremely high peak intensity allows high-quality micro- and nanofabrication of three-dimensional (3D) structures. This unique capability opens up a new route for fabrication of microfluidic sensors for biochemical applications. The present paper presents a comprehensive review of recent advancements in femtosecond laser processing of glass for a variety of microfluidic sensor applications. These include 3D integration of micro-/nanofluidic, optofluidic, electrofluidic, surface-enhanced Raman-scattering devices, in addition to fabrication of devices for microfluidic bioassays and lab-on-fiber sensors. This paper describes the unique characteristics of femtosecond laser processing and the basic concepts involved in femtosecond laser direct writing. Advanced spatiotemporal beam shaping methods are also discussed. Typical examples of microfluidic sensors fabricated using femtosecond lasers are then highlighted, and their applications in chemical and biological sensing are described. Finally, a summary of the technology is given and the outlook for further developments in this field is considered.

## 1. Introduction

Microfluidic sensors are devices that allow precise control and manipulation of fluids that are geometrically constrained to regions with typically sub-millimeter dimensions, or that can handle extremely small fluid volumes down to picoliters. Typically, fluids are transported, mixed, separated or processed *via* passive fluid control techniques using microfluidic channels, while components such as micro-pumps or micro-valves are required for some active microfluidic devices [1,2]. Using these devices, conventional processes normally carried out in a lab can be miniaturized and performed on a single chip. This leads to enhanced efficiency and portability, and also reduces the volume of sample and reagent required when performing multilevel assessments involving, for example, chemical, biological and medical analyses. The most significant benefits of these devices are a scaling down of the size, minimal consumption of reagents, reduced manufacturing costs, and enhanced detection speed and sensitivity. Due to their high portability and sensitivity, these devices have become powerful detection and analysis tools for a broad range of applications including biomedical research, healthcare, pharmaceuticals, environmental monitoring, and homeland security. Moreover, there is also the possibility of further enhancing the performance of microfluidic devices by monolithically integrating electronic, mechanical or optical capabilities [3,4].

Until now, microfluidic sensors have mainly been manufactured using soft lithography, which is carried out using the optically transparent, soft elastomer polydimethylsiloxane (PDMS). Although this technique is rapid and cost effective, it requires additional stacking and bonding processes in order to fabricate 3D microfluidic structures in transparent substrates. Other conventional methods for producing microfluidic systems include planar microfabrication techniques such as injection molding and semiconductor processes based on photolithography, both of which also require stacking and bonding in order to construct 3D structures. Furthermore, the above techniques have encountered considerable challenges with regard to monolithic integration of multiple functionalities, for which 3D fabrication approaches are typically needed. During the past two decades, femtosecond laser microfabrication has been shown to be a highly attractive solution for 3D manufacturing of transparent materials [5,6]. It exhibits great promise for the fabrication of microfluidic, photonic, micro-optical, microelectronic, and micromechanical components. Its unique capability for 3D integration of functional microcomponents makes it a powerful state-of-the-art micromachining tool, in particular for fabrication of microfluidic sensors. Here, we demonstrate how femtosecond laser microfabrication can be used to create innovative microfluidic systems for a wide variety of sensing applications.

This review article mainly focuses on recent advancements in femtosecond laser fabrication of microfluidic sensors in glass. The remainder of this article is organized as follows. In Section 2, the characteristics of femtosecond laser processing are discussed. In Section 3, the experimental setup used for femtosecond laser direct writing (FsLDW) is described, including advanced beam and pulse shaping methods. Section 4 provides an overview of femtosecond laser processing of different glass for microfluidic applications. The main part of this article, Section 5, discusses a variety of sensor applications based on femtosecond laser fabrication, including 3D integration of microfluidic, nanofluidic, optofluidic, electrofluidic, and surface-enhanced Raman-scattering (SERS) devices, in addition to fabrication of devices for microfluidic bioassays and lab-on-fiber (LOF) devices. Finally, a summary is given in Section 6, which also discusses the future outlook.

## 2. Characteristics of Femtosecond Laser Processing

Laser pulses with time durations of a few femtoseconds (1 fs = 10^−15^ s) to several hundred femtoseconds are referred to as femtosecond pulses. Such pulses have a broad optical spectrum (e.g., a 40-fs pulse with a center wavelength of 800 nm typically has a spectral width of about 30 nm), and can be generated using mode-locked oscillators. Amplification of ultrashort pulses typically requires a technique referred to as chirped pulse amplification in order to avoid damage to the gain medium of the amplifier [7]. Although femtosecond laser processing was first performed in 1987 [8,9], since the 1990s, when the development of Ti:sapphire lasers with a regenerative amplifier system revolutionized ultrafast laser science, the intense femtosecond laser pulses that they produce have been increasingly used for materials processing [10–12]. Currently, femtosecond laser processing is not only a subject of intensive academic research but also a reliable tool for both practical and industrial applications [13–15].

**Figure 1.**
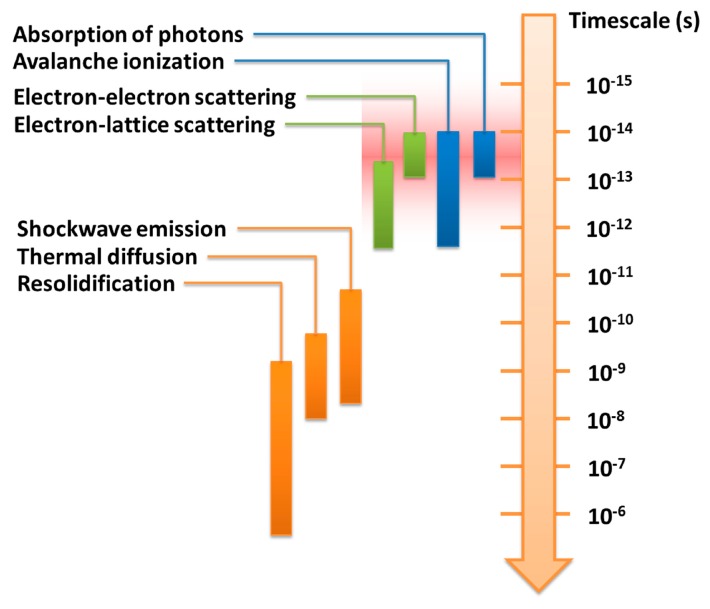
Typical timescales during interaction of ultrashort laser pulses with transparent materials.

Fundamental investigations into laser processing of materials have revealed that the time dependence of ultrafast electron and lattice dynamics is quite different for long and for ultrashort laser pulses. It was established that intense laser pulses with durations of nanoseconds can be used to anneal the lattice in a manner of thermal processing. For ultrashort laser pulses in the femtosecond regime, however, the electrons and the lattice are driven far out of equilibrium and disordering of the lattice can occur. During the interaction of an ultrashort pulse with a transparent material, the laser energy is first deposited into the electrons and then transferred to the lattice, with several regimes of electron excitation and relaxation [5,16]. These regimes and the timescales of the corresponding processes are shown in Figure 1.

**Figure 2.**
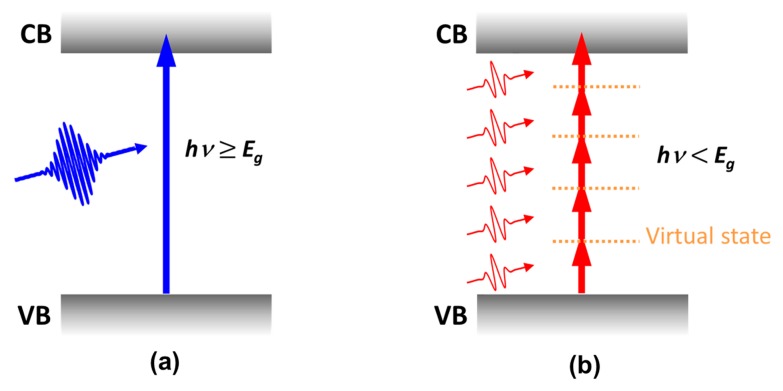
Schematic illustration of (**a**) single- and (**b**) multiphoton absorption process. CB: conduction band, VB: valence band.

The interaction of femtosecond laser pulses with transparent materials with wide band gaps (e.g., 800-nm femtosecond pulses interacting with fused silica glass) is generally regarded as a multiphoton absorption (MPA) process. When a single photon has an energy exceeding the band gap of the material, it is absorbed by exciting an electron from the valence band (VB) to the conduction band (CB) by single-photon absorption, as illustrated in Figure 2a. However, when an extremely high density of photons (*i.e.*, extremely high-intensity light) is incident on the material, an electron can be excited by multiple photons *via* virtual states, even if the photon energy is smaller than the band gap, as shown in Figure 2b. Due to the nonlinear nature of MPA, tightly focused femtosecond laser pulses can be strongly absorbed even by a transparent medium. This light-matter interaction occurs only near the focal volume where the peak intensity is sufficiently high to induce MPA with a large absorption cross section. This is fundamentally different to the possible avalanche ionization (AI) process that can induce breakdown in the case of interaction of long laser pulses (typically nanoseconds) with transparent materials [17]. For the long pulses, such AI dominated processes are mainly caused by impurities and dislocations in the focal volume. They therefore occur stochastically and are irreproducible. In contrast, the MPA dominated process induced by femtosecond lasers is deterministic in that it is only seeded by multiphoton/tunnel ionization. This unique characteristic allows reproducible and controllable processing to be performed using femtosecond lasers.

**Figure 3.**
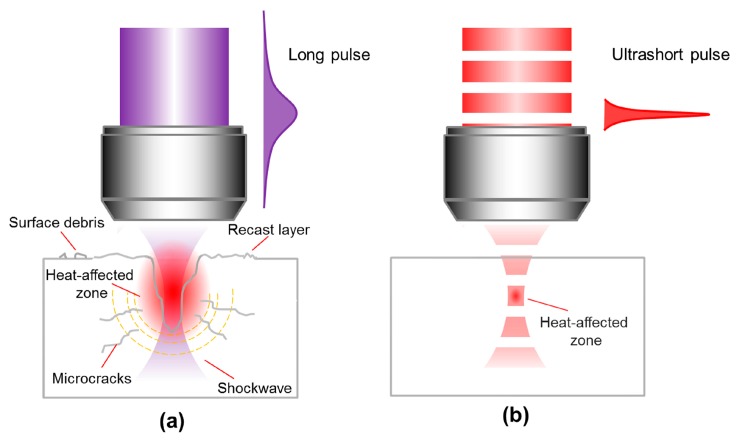
Laser processing of transparent material using (**a**) long pulses and (**b**) ultrashort pulses.

**Figure 4.**
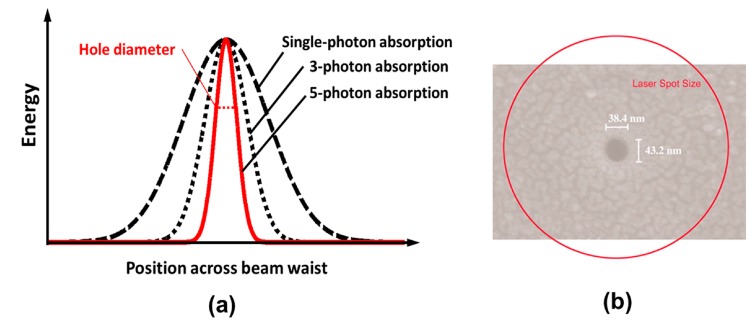
(**a**) Profiles of energy absorbed during single-photon absorption (black dashed line), 3-photon absorption (black dotted line) and 5-photon absorption (red solid line). The hole diameter is indicated by the red dashed line; (**b**) SEM micrograph of nanometer-scale hole fabricated in glass. The red circle indicates the 1/e^2^ focus-spot size [19] (Reproduced with permission from PNAS. ©2004 by the National Academy of Sciences, Washington, DC, USA).

Figure 3 schematically illustrates the interaction of long and ultrashort laser pulses with a transparent material. Compared with conventional laser fabrication techniques that use continuous wave (CW) lasers or lasers with nanosecond pulses, femtosecond laser processing has several distinct advantages:

(1) *Elimination of thermal diffusion*. The use of a focused femtosecond laser beam can significantly reduce thermal effects during material processing, due to the fact that most of the energy associated with the pulse can be deposited into electrons before being transferred to the lattice when the pulse duration is shorter than the electron-phonon coupling time (typically several to a few tens of picoseconds), as indicated by the red shaded region in Figure 1. The free-electron temperature increases rapidly and becomes much higher than the lattice temperature, a process that can be characterized by a two-temperature model [18]. Material in the laser interaction region is then ejected in a very short time in the form of hot dense plasma, leaving the local lattice still “cold”. This effect is obvious for femtosecond lasers with a low repetition rate (a few kHz or less), even for a high repetition rate (a few hundred kHz or more), such a process can effectively suppress the formation of a heat-affected zone (HAZ), thus enabling the fabrication of fine structures with micro- or nanoscale features. On the other hand, for laser pulse durations significantly longer than the electron-phonon coupling time, although the radiation energy is first transferred to the electrons, the electrons transfer it to the lattice before the pulse has terminated. This allows the electrons and the lattice to reach an equilibrium state, and the laser simply “heats” the solid to its melting temperature within the duration of the pulse. This give rises to significant thermal diffusion, and consequently a reduction in fabrication quality and achievable resolution.
(2) *Internal processing*. Another important benefit of femtosecond laser microfabrication is that it can be used to machine transparent materials in a 3D space-selective manner, because MPA only occurs in the beam focal volume, where the light intensity is sufficiently high. It is noteworthy that this unique 3D capability cannot be achieved using nanosecond UV lasers since when single photon absorption is involved, modification always begins at the surface and moves inwards, as illustrated in Figure 3a. Even if a nanosecond laser with a wavelength sufficiently long for the material to be transparent is used, 3D processing still cannot be realized because a radiation intensity sufficiently high to induce MPA will also lead to strong thermal effects such as cracking, due to the long pulse duration. The ability to create arbitrary 3D structures within transparent substrates by the use of femtosecond lasers enables the production of novel device architectures with enhanced functionality and improved compactness.
(3) *Sub-diffraction-limited resolution*. Again due to the characteristics of MPA, the effective processing profile of a femtosecond laser beam can be much narrower than its Gaussian intensity profile, as indicated in Figure 4a. This is because the distribution of absorbed energy for *N*-photon absorption is proportional to the *N*th power of the laser intensity (*N* is the number of photons simultaneously absorbed by the material to produce a transition from the VB to the CB). The effective beam diameter is then given by ω_0_/N^1/2^, where ω_0_ is the actual beam diameter. Meanwhile, suppression of the HAZ by femtosecond laser processing restricts the laser-modified region to within a few nanometers of the volume where the laser fluence is above the threshold for inducing MPA. This makes it easy to achieve a fabrication resolution far beyond the optical diffraction limit by adjusting the intensity at the center of the Gaussian beam profile so that it is as close as possible to the threshold intensity for modification. For example, a resolution of λ/20 has been realized for both surface modification [19] (see Figure 4b) and internal modification [20], and a resolution of λ/40 has been reported for a two-photon polymerization (TPP) process [21].
(4) *Multifunctional integration*. The tightly focused femtosecond laser beam can be used to precisely modify or even permanently change the physical and/or chemical properties of the material in a spatially selective manner. A host of exiting novel effects, including surface/bulk microstructuring, subtractive micromachining, refractive index modification, selective metallization, and TPP, can be achieved by FsLDW, which enables multifunctional integration in a single substrate.

Owning to the above advantages, femtosecond laser processing has attracted significant attention and promises a wide variety of intriguing applications in not only integrated photonic devices (e.g., waveguide writing [11], polarization sensitive optics [22], quantum circuits [23].) but also integrated microfluidic and optofluidic systems (e.g., lab-on-a-chip (LOC) devices, micro-total analysis systems) [24,25]. In addition, femtosecond laser irradiation of transparent materials such as glass and crystals has led to many other novel phenomena, such as the formation of nanovoids [26] and periodic nanogratings [27], element redistribution [28], nanocrystallization [29], and optical “quill” writing [30].

There are already a number of review papers available, with emphases on the general characteristics of femtosecond laser micromachining [5,6,31], the fundamental physics [32–34], basic processing techniques [35–37] and innovative applications including photonic devices [38–44], microfluidics and optofluidics [24,25,45–47], surface and bulk micro- and nanostructuring [48–51] TPP [52–58], and ultrafast laser surgery [59–61]. However, a review article focusing on fabrication of various kinds of sensors in glass, which is the topic of the present article, has not appeared before now.

## 3. Experimental Setup for Femtosecond Laser Direct Writing

**Figure 5.**
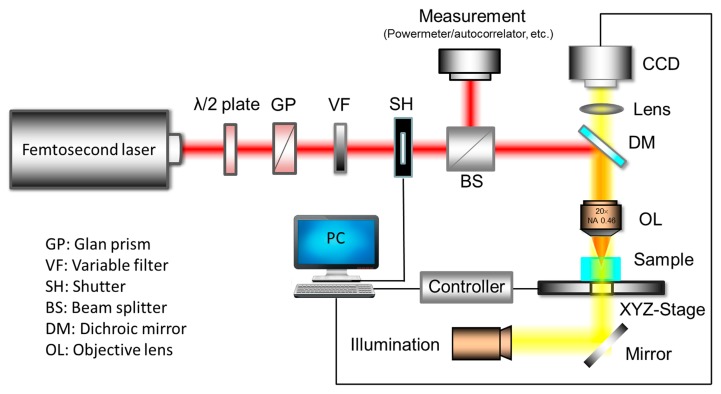
Schematic configuration of general femtosecond laser direct writing (FsLDW) system.

Figure 5 shows a schematic diagram of a typical FsLDW system. Generally, a femtosecond amplifier system is required to process glass. A λ/2 plate in combination with a Glan prism is used to coarsely adjust the laser power, while an additional variable neutral density (ND) filter is used to tune the laser power more precisely. The exposure time is controlled by a mechanical shutter. To monitor the stability of the input laser power during fabrication, a beam splitter is employed to split the input laser beam into two with a fixed intensity ratio. One of the split beams is monitored using a power meter, and an autocorrelator is used to evaluate the pulse duration [35]. In the general case, the second split beam is passed through a microscope objective to produce a tightly focused spot. For special purposes, however, other types of optical elements can be used, such as a cylindrical lenses for line scanning during high-volume processing [62], or an axicon for producing a non-diffractive beam for high-aspect-ratio drilling [36]. The sample is placed on a high-precision XYZ translation stage controlled by a computer for 3D translation. A dichroic mirror and a charge-coupled device (CCD) camera connected to the computer are installed above the focusing lens to enable the fabrication process to be monitored in real time.

In femtosecond laser processing of transparent material, laser conditions such as the spatiotemporal beam profile [63], repetition rate [64], scanning speed [65], and polarization [27] can have significant impacts on the fabricated microstructure. These parameters can be precisely tuned using electrical and optical devices such as those shown in Figure 5. A combination of a wave plate and a polarizer can be used to control the polarization of the laser beam, which is important for some types of applications [66,67]. An ND filter or an attenuator can be used to produce inhomogeneous illumination, which can lead to a surprising “quill” writing effect [68]. Using a programmable shutter or an external acousto-optic modulator, a burst of pulses can be produced for single-step writing of fiber Bragg grating (FBG) structures [69]. To compensate for the low throughput of conventional FsLDW by employing a point-by-point scanning method, a microlens array or a diffractive optical element can be employed to achieve parallel processing [70,71].

**Figure 6.**
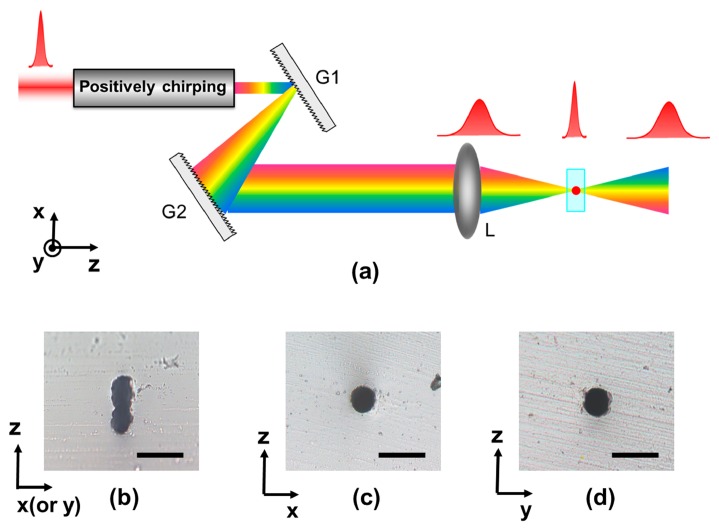
(**a**) Schematic diagram of spatiotemporal focusing for materials processing. The images are cross-sectional optical micrographs of microfluidic channels fabricated in fused silica by FsLDW with (**b**) conventional focusing (XZ or YZ plane); (**c**) spatiotemporal focusing (XZ plane) and (**d**) spatiotemporal focusing (YZ plane) [76] (Reproduced with permission from OSA. ^©^2010 by the Optical Society of America). The scale bars in (**b**–**d**) represent 50 µm.

Most FsLDW processes employ transverse direct writing, in which the writing direction is perpendicular to the laser propagation direction. For commercially available objective lenses, there is a tradeoff between the fabrication resolution and the working distance for commercially available objective lenses, the focal volume produced by the objective lens is generally elongated along the beam direction when a relatively long working distance is used in order to process deep regions in the bulk material, resulting in different lateral and axial resolutions. This causes the cross-sectional shape of microfluidic channels to be elliptical, which is not suitable for many microfluidic applications. Efforts have been made to solve this problem [72–75], and the most simple and widely employed approach is beam shaping using a slit. For instance, if a narrow slit with a width of about 500 µm is placed directly above a 20× objective lens, and the laser writing direction is parallel to the slit direction, microstructures with symmetrical cross sections can be produced. More recently, a technique referred to as spatiotemporal focusing has been proposed for achieving isotropic resolution during femtosecond laser microfabrication [76–78]. The key idea is that by focusing a spatially disperse pulse, the pulse duration increases with distance from the focal plane, causing a significant reduction in the peak intensity in out-of-focus regions. Figure 6a shows a schematic diagram of this technique, in which a single-pass compressor G1–G2 is used to disperse the spectral components of the input pulse in space, before which the pulse is pre-chirped to compensate for the negative chirp induced by G1–G2. Recombination of the spectral components is then achieved by a lens L, causing the shortest pulse duration to occur at the focus. Figure 6b–d show cross-sectional optical micrographs of microfluidic channels in fused silica fabricated using FsLDW with conventional focusing and spatiotemporal focusing. The effectiveness of the shape control method can be clearly seen.

## 4. Femtosecond Laser Processing of Glass

Glass is considered to be a good candidate for substrates for microfluidic sensors fabricated by 3D femtosecond laser microfabrication because of its wide optical transparency, low thermal expansion, and good chemical inertness and biocompatibility. Although a wide variety of glass have been explored for femtosecond laser processing (particularly for optical waveguide writing) [11,79–81], fused silica and a kind of photosensitive glass termed Foturan are the most common materials used for microfluidic sensors, since they exhibit a selective chemical etching ratio of 30:1 or greater in HF acid and other solutions following laser processing.

**Figure 7.**
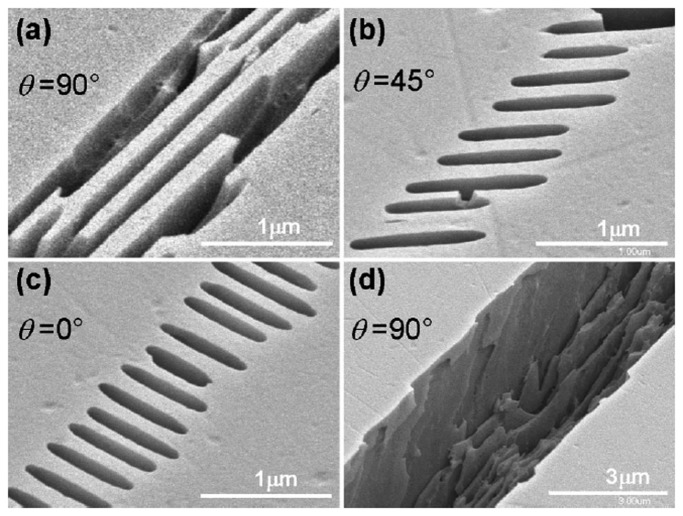
(**a**–**c**) Top-view SEM images of long-range periodic nanostructures formed along the writing direction for different values of θ (angle between laser-beam polarization direction and writing direction) for a pulse energy *E_p_* = 300 nJ; (**d**) *E_p_* = 900 nJ. The structures were revealed after 20 min of etching in a 0.5% aqueous solution of HF [83] (Reproduced with permission from OSA. ^©^2005 by the Optical ciety of America).

**Figure 8.**
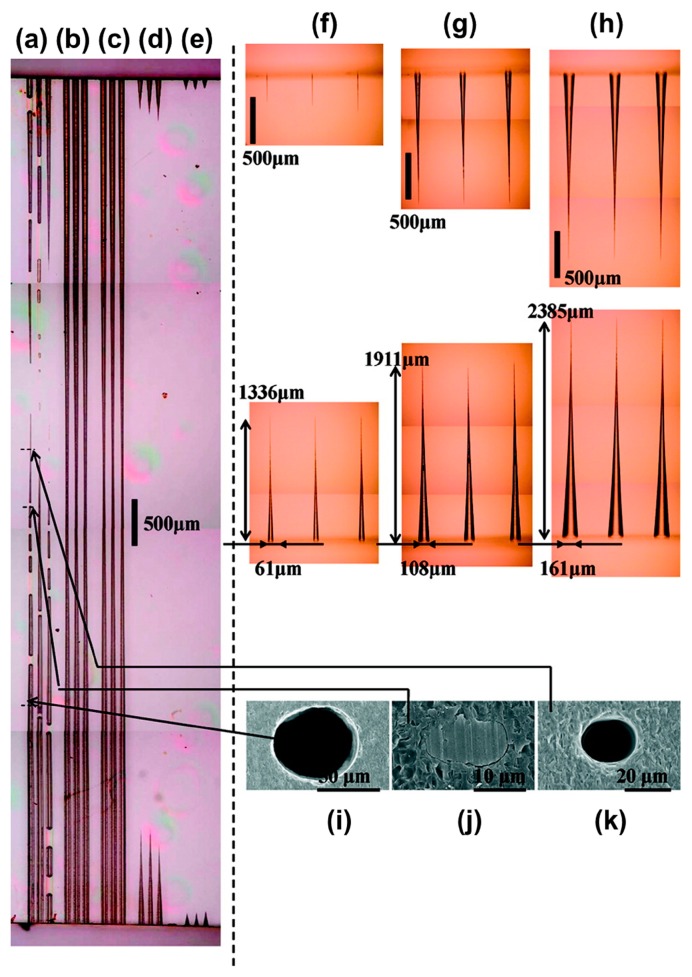
Reflectance optical micrographs of etching profiles in silica glass substrate (9.2 mm × 10 mm × 0.5 mm) using 10 M (35.8%) aqueous KOH (20 mL) and 2.0% aqueous HF (20 mL). The optical micrograph on the left shows channels fabricated after soaking for 60 h in KOH at 80 °C, following irradiation using femtosecond pulse trains (interval: 0.1 μm) through a 40× objective lens (NA: 0.65), 10 μm below the surface at different laser powers of (**a**) 500; (**b**) 400; (**c**) 300; (**d**) 200; and (**e**) 100 nJ/pulse. The optical micrographs on the top-right show the time evolution of channel formation in aqueous HF at ambient temperature (both ends of the channels shown). The specimen was irradiated with a laser power of 360 nJ/pulse through a 40× objective lens (NA: 0.65) and submerged in the solution for (**f**) 24; (**g**) 48; and (**h**) 72 h. The FE-SEM images on the bottom right show cross sections of the leftmost channel in (a) at the positions indicated. In (**i**) and (**k**); the channels are open, whereas in (**j**); the channel is still filled with precipitates [85] (Reprinted with permission from ACS. ^©^2009 by the American Chemical Society).

The fabrication procedure for microfluidic structures in fused silica generally involves FsLDW followed by wet chemical etching [82]. The laser-modified regions exhibit an enhanced etching rate, which is thought to be due to a reduction in the Si–O–Si bond angle, although this is still a subject for debate. Further investigation has shown that the etching selectivity depends on the polarization of the laser beam. As shown in Figure 7, a linearly polarized laser beam can cause spatial modulation of the absorbed laser energy with a nanoscale periodicity, in the direction perpendicular to the polarization direction. Thus, scanning the laser along its polarization direction enhances the etching selectivity [27,83,84]. The most commonly used etchant has been HF, which offers a selective etching ratio of up to about 30:1. However, it was recently found that very highly selective etching could be achieved using KOH, allowing for fabrication of microfluidic channels with an aspect ratio of about 200:1, as shown in Figure 8, although the etching rate is extraordinary low compared to that for HF [85,86]. Generally, the etched structures exhibit a surface roughness of a few tens of nanometers, thus hampering their usability for many applications. To solve this problem, several post-processing heat treatments, including oxygen/hydrogen flame polishing [87,88], annealing in an oven [89], and CO_2_ laser reflow [90] have been proposed. Using these methods, the surface roughness can be substantially reduced to a few nanometers, opening up new possibilities for the design of useful devices. A host of on-chip optical elements such as microlenses, hollow optical waveguides, and optical micro-resonators, has been developed in this way [87–90]. However, it should be noted that only the oven annealing method can be used to smooth the internal walls of hollow microstructures, which are not accessible to any mechanical polishing tools. Temperatures as high as about 1200 °C are required when annealing fused silica [89].

Foturan glass exhibits similar selective etching characteristics, but the mechanism is quite different to that for fused silica [91]. The selective etching ratio enhancement in Foturan glass is the result of a photochemical reaction, while in fused silica, it is caused by a photophysical process. More specifically, femtosecond laser irradiation of Foturan glass reduces Ag dopant ions to Ag atoms due to the generation of free electrons by MPA, and during subsequent annealing, Ag nanoclusters are formed by diffusing Ag atoms. These nanoclusters then act as seeds for the formation of crystalline lithium metasilicate, which has an etching rate in HF that is about 50 times higher than that of the unirradiated glass matrix. Thus, the typical method for fabricating microfluidic structures in Foturan glass involves FsLDW, heat treatment in a programmable oven, and wet chemical etching in an ultrasonic bath. For Foturan glass also, an additional post-etching annealing step is required for surface smoothening. However, an annealing temperature of about 570 °C is sufficient, which is significantly lower than that for fused silica. This is due to the much lower melting temperature of Foturan [47]. In this way, optical elements can be realized in Foturan glass. Details concerning characteristics of Foturan, fabrication techniques and applications can be found in several review articles [45–47].

As an alternative to wet chemical etching, 3D microfluidic structures can also be fabricated in glass by femtosecond laser 3D drilling from the rear surface while the substrate is immersed in distilled water, a technique that is frequently referred to as liquid-assisted femtosecond laser drilling [92,93]. The water introduced into the microchannels can help remove ablation debris, greatly increasing the drilling length compared to that achievable by drilling in ambient air. Since this approach does not rely on etch selectivity, it is easier to implement and is more environmentally friendly. More importantly, it can be applied to any material that is transparent to the writing pulses. However, even with the assistance of water, ablation debris still clogs the microchannel when the depth or length of the channel is several hundred micrometers, restricting the size of fabricated microstructures.

To overcome this issue, a novel porous glass was proposed as an exciting new substrate material for fabrication of micro- and nanofluidic structures [20,94–96]. It is produced by removing the borate phase from phase-separated alkali-borosilicate glass in a hot acid solution. The composition of the porous glass is approximately 95.5SiO_2_-4B_2_O_3_-0.5Na_2_O (wt. %). The pores have a mean diameter of about 10 nm, and are uniformly distributed in the glass, occupying about 40% of its volume. These pores form a 3D connective network which allows liquid to flow through the entire glass volume. Interestingly, by annealing the glass substrate at about 1150 °C, the mesoporous glass can be consolidated and the pores sealed. Compared with fused silica and Foturan, this glass offers the advantage of enabling fabrication of 3D microfluidic channels with arbitrary geometries and unlimited lengths, with feature sizes far beyond the optical diffraction limit. In addition, the fabrication procedure is simple and contamination-free, involving only femtosecond laser ablation in water and subsequent annealing to seal the pores. A range of intriguing applications of this porous glass for femtosecond laser microfabrication will be discussed in Section 4.

All of the glass discussed so far can also be used in additive processes such as selective metallization [97–99] and TPP [100–102]. With Foturan (and other types of novel metal-doped photosensitive glass), selective metallization can be achieved by only the two steps of FsLDW followed by electroless metal plating. The Ag clusters produced during FsLDW on the substrate surface can act as catalysts for the chemical reactions involved in electroless plating; the surface roughening that results from laser ablation also promotes anchoring of the plated metal [98]. In contrast, when performing electroless metal plating of fused silica (or other insulators), it is necessary to first deposit a silver nitrate seed layer on the surface [97]. Selectively metallized structures can be used for applications such as optical modulation and electrofluidics [99], SERS sensors [98], and plasmonics devices [100]. Meanwhile, TPP can create functional 3D polymer microcomponents in glass substrates using an additive manufacturing process [101,102]. Generally, an inverted microscope configuration is employed in the TPP process, while an upright structure is sometimes required for fabrication of hybrid components.

**Figure 9.**
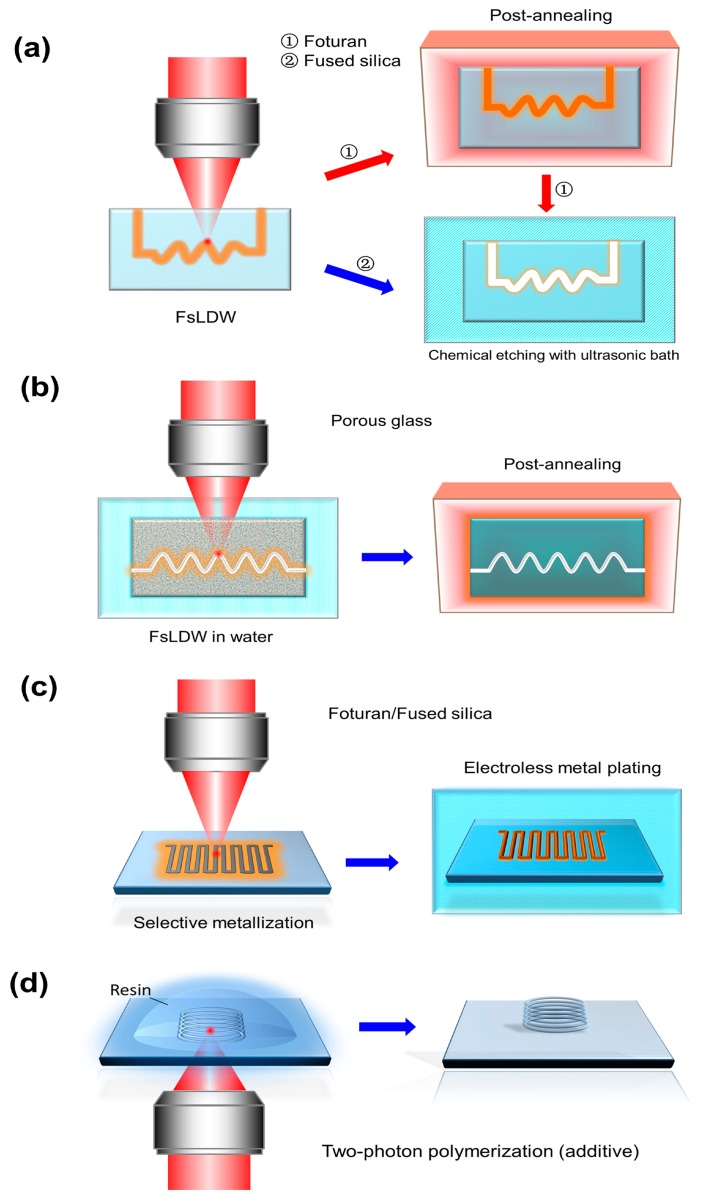
Schematic diagrams of fabrication procedures for (**a**) microfluidic structures in Foturan or fused silica glass; (**b**) micro- or nanofluidic structures in porous glass; (**c**) microelectronic structures on glass; and (**d**) 3D polymer micro- and nanostructures on glass substrates by two-photon polymerization (TPP).

The above additive processes enable selective metallization and polymerization of not only the surface of planar substrates or fiber tips, but also the bottom or sidewalls of open microfluidic structures and even closed microfluidic channels [101,103]. In addition, the combined use of additive and subtractive techniques allows new advanced functionalities to be incorporated for a wide range of sensor applications. Figure 9 shows schematic diagrams of FsLDW based procedures for fabricating 3D micro- and nanofluidic devices, microelectronic components, and 3D polymer micro- and nanostructures.

**Figure 10.**
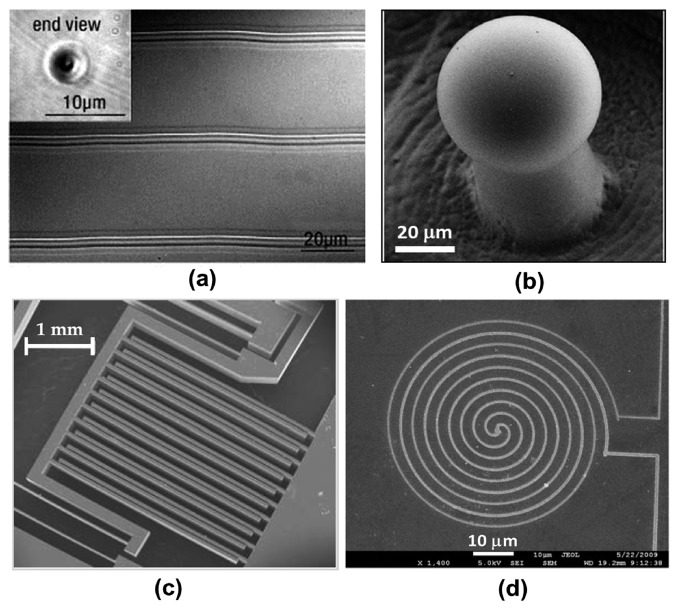
(**a**) Optical micrograph of an oscillator-only femtosecond-laser-micromachined waveguide inside bulk glass. The inset shows one end-face of the waveguide [80] (Reproduced with permission from OSA. ^©^2001 by the Optical Society of America); (**b**) SEM image of a spheroidal optical microcavity fabricated in glass using FsLDW [104] (Reproduced with permission from OSA. ^©^2013 by the Optical Society of America); (**c**) SEM image of a micro-actuator fabricated from a single piece of fused silica by femtosecond laser micromachining combined with chemical etching [105] (Reproduced with permission from AIP. ^©^2012 by the American Institute of Physics); (**d**) SEM image of a patterned silver microcircuit on a glass substrate produced by femtosecond-laser-induced electroless plating [106] (Reproduced with permission from Wiley. ^©^2010 Wiley-VCH Verlag GmbH & Co. KGaA, Weinheim).

As mentioned above, monolithically integrated microfluidic sensors commonly contain multiple components with different functionalities, such as photonic circuits, optical components, micromechanical components and electronic components. Due to the powerful manufacturing capability of FsLDW, these components can be produced in a very similar manner. Typical examples are shown in Figure 10. The fabrication of an optical waveguide was one of the first demonstrations of the potential of femtosecond laser micromachining for photonic applications. Figure 10a shows optical waveguides written inside bulk Corning 0211 glass by translation of the sample at 20 mm/s in the direction perpendicular to an incident 25-MHz train of 5-nJ, sub-100-fs laser pulses focused with a 1.4-NA objective [80]. Each cylinder forms a single-mode optical waveguide, indicating that the refractive index is larger at the center than in the surrounding material. It is noteworthy that the pulse energy required for fabricating such waveguides can be as low as a few nanojoules, requiring only a high repetition-rate oscillator, which means that high-throughput fabrication can be achieved. Figure 10b shows a whispering-gallery-mode microlaser fabricated in a Nd:glass chip by FsLDW. The main fabrication procedure involves the creation of freestanding microdisks supported by thin pillars, by femtosecond laser ablation of a glass substrate immersed in water, followed by CO_2_ laser annealing for surface smoothing [104]. The quality (Q) factor for the fabricated microcavity was measured to be 1.065 × 10^6^. Lasing was observed at a pump threshold as low as ∼69 µW at room temperature with a continuous-wave laser diode operating at 780 nm. As an example of an optomechanical device, a comb drive glass actuator manufactured entirely by a femtosecond laser is shown in Figure 10c. It consists of two elements: a comb, formed by two intertwined sets of parallel beams, and a flexure made of four leaf springs. Using optically transparent indium tin oxide (ITO) electrodes, each set of parallel beams is placed under a different electrical potential so that a capacitor is formed. Under an applied voltage, a net electrostatic force causes the mobile element to move downward. Such actuators are common in applications such as adaptive optics and microscopy, in addition to being used for integration of all-optical devices and transparent microelectromechanical systems (MEMS) [105]. Figure 10d shows an example of a microheater component fabricated using femtosecond-laser-induced electroless plating [106]. By careful control of the laser power, the line width of the nanowires can be finely adjusted in the range 125 to 500 nm. Any desired circuit or wire pattern can be directly written on many types of non-planar substrates, such as rough bases, spherical surfaces, or even sharp corners. Moreover, the patterned silver nanowires were found to have a low resistivity of about 1.6 × 10^−7^ Vm, which highlights their potential for circuitry and electrical interconnections.

Typical properties and applications of the three kinds of glass are listed in Table 1.

## 5. Fabrication of Integrated Microfluidic Sensors

Compared with conventional 3D microfluidic prototyping techniques, such as the standard lithographic methods used in the semiconductor industry, FsLDW allows direct creation of complex 3D microfluidic structures with almost arbitrary configurations, without the need for complicated stacking or bonding procedures. Moreover, this technique is clearly outpacing other fabrication technologies with regard to monolithic integration of advanced functionalities such as photonics, micro-optics, microelectronics, and micromechanics into microfluidic structures. Here, we demonstrate how FsLDW can be used to introduce multiple functionalities to a range of microfluidic sensor applications.

### 5.1. Fabrication of True 3D Micro- and Nanofluidic Structures

**Figure 11.**
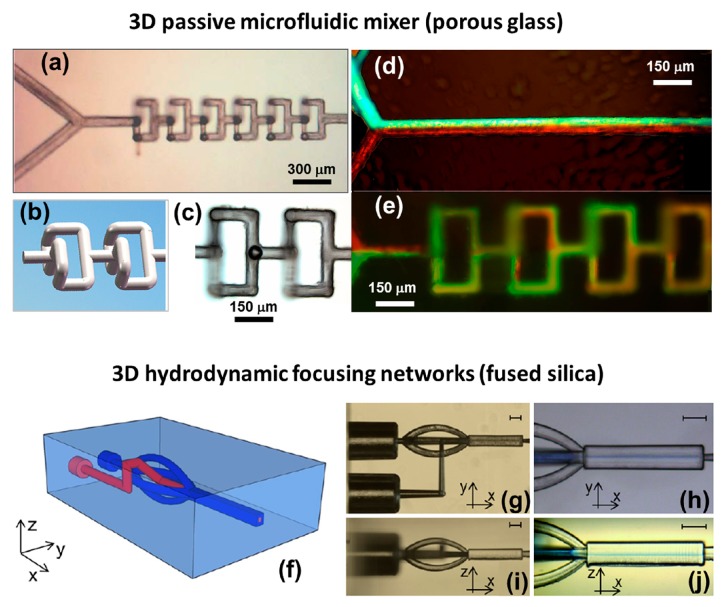
Fabrication of a 3D passive microfluidic mixer in porous glass, and 3D hydrodynamic networks in fused silica using FsLDW, respectively. (**a**) Optical micrograph of microfluidic mixer; (**b**) Schematic illustration of two mixing units; (**c**) Top-view optical micrograph of two mixing units. Fluorescence microscopy images of (**d**) 1D and (**e**) 3D microfluidic mixing experiments (Reproduced with permission from RSC. ^©^2012 by the Royal Society of Chemistry); (**f**) Schematic illustration of 3D hydrodynamic focusing network [95]; (**g**) Top-view and (**i**) side-view optical micrographs of fabricated device; (**h**) Top-view and (**j**) side-view optical micrographs of flow confinement in the horizontal and vertical directions achieved with a sample/sheath pressure ratio of 0.55. The scale bars in (**g**–**j**) represent 100 µm [120]. (Reproduced with permission from RSC. ^©^2014 by the Royal Society of Chemistry).

Although various microfluidic structures can be fabricated in fused silica or Foturan glass using a combination of FsLDW and chemical etching, promising a broad range of important applications in biochemistry analysis [24,25], it is still challenging to prepare microfluidic structures with arbitrary aspect ratios, or to fabricate 3D nanofluidic structures in this manner, since the selective etching ratio is limited. Recently, a new strategy has been developed for fabricating microchannels of almost unlimited lengths and arbitrary geometries. This involves FsLDW in mesoporous glass immersed in water followed by annealing, and has allowed the successful fabrication of long square-wave-shaped microchannels and large microfluidic chambers [94–96]. In particular, a passive microfluidic mixer was constructed using 3D microchannels written in mesoporous glass, which exhibited superior mixing efficiency to its 1D counterpart (see Figure 11a–e). Figure 11a presents an optical micrograph of the micromixer, which consists of six mixing units. The length of the horizontal and vertical channels in each mixing unit is 150 µm, as shown in Figure 11b (schematic view) and Figure 11c (plan-view optical micrograph). Figure 11d and e respectively show experimental results for mixing of two fluorescent dye solutions (fluorescein sodium and Rhodamine B) in 1D and 3D microfluidic mixers fabricated using the same direct writing conditions. With the 3D mixer, after passing through three mixing units (a distance of 0.9 mm), corresponding to a mixing time of about 10 ms, the two fluids were well mixed. In contrast, with the 1D microfluidic channel, efficient mixing was not achieved even after a propagation distance of about 1300 µm.

More interestingly, the use of FsLDW allowed fabrication of true nanochannels by combining the so-called “threshold effect” with the formation of a periodic nanograting [20]. Such a periodic grating can be formed in glass when it is irradiated with a linearly polarized femtosecond laser beam, which implies that the absorbed energy is spatially modulated with a nanoscale periodicity at the focal spot. When the laser intensity is intentionally reduced so that the threshold value is exceeded only within the central region of the focal volume, a single cycle of the modulated energy distribution can be used to induce ablation. Using this approach, fabrication of a channel with a width of about 40 nm and a length of about 40 µm (aspect ratio of about 1000:1) was realized.

It was also recently reported that using femtosecond laser irradiation followed by chemical etching, complicated 3D microfluidic networks with large feature sizes could be fabricated in fused silica substrates, which are suitable for 3D hydrodynamic focusing and high-efficiency cell sorting [120]. The design of the 3D-focusing system is shown schematically in Figure 11f–i respectively show top- and side-view optical micrographs of the fabricated prototype. Hydrodynamic focusing can be easily implemented by controlling just two independent pressures. In this device, 3D symmetric flow confinement of particles within a narrow region near the center of the channel can be achieved. Figure 11h and j show top- and side-view images of the device during operation. To increase the visibility of the confined flow, a blue-colored fluid was used for the sample, and distilled water was used for the sheath flow. The width of the sample flow region is about 10 µm, both in the horizontal and vertical cross sections, at a sample/sheath pressure ratio of 0.55. By further integrating optical waveguides into the system by femtosecond laser modification, a monolithic cell counter was constructed, with a counting rate of up to 5000 cells/s.

### 5.2. Optofluidic Sensors

Optofluidics refer to a class of optical systems that are synthesized by the marriage of microfluidics and optics/photonics. They exhibit tunable optical properties and physical adaptability, and show high potential for lab-on-a-chip (LOC) and bio-photonics applications [3,4,121]. This field emerged in the 2000 s when both microfluidics and nanophotonics were maturing, and their synergy became feasible. Although the primary fabrication techniques for optofluidics are based on lithographic methods, FsLDW has been shown to be a promising alternative for flexible fabrication of true 3D multifunctional structures [24,25]. Currently, fabrication of optofluidic structures by FsLDW can be achieved by incorporation of optical waveguides and/or micro-optical components into microfluidic systems. Using a unified process, a wide variety of sensing devices such as a Mach-Zehnder interferometer (MZI) [108], single-cell detection and manipulation systems [109,122,123], and photonic microcomponents such as microfluidic lasers [117] and waveguides [124] can be fabricated in both fused silica and Forturan glass substrates. It is noteworthy that integration of microfluidic structures with optical waveguides in porous glass has not yet been demonstrated. However, this is now being attempted by writing waveguides in consolidated porous glass in which the embedded microfluidic structures have been formed beforehand.

**Figure 12.**
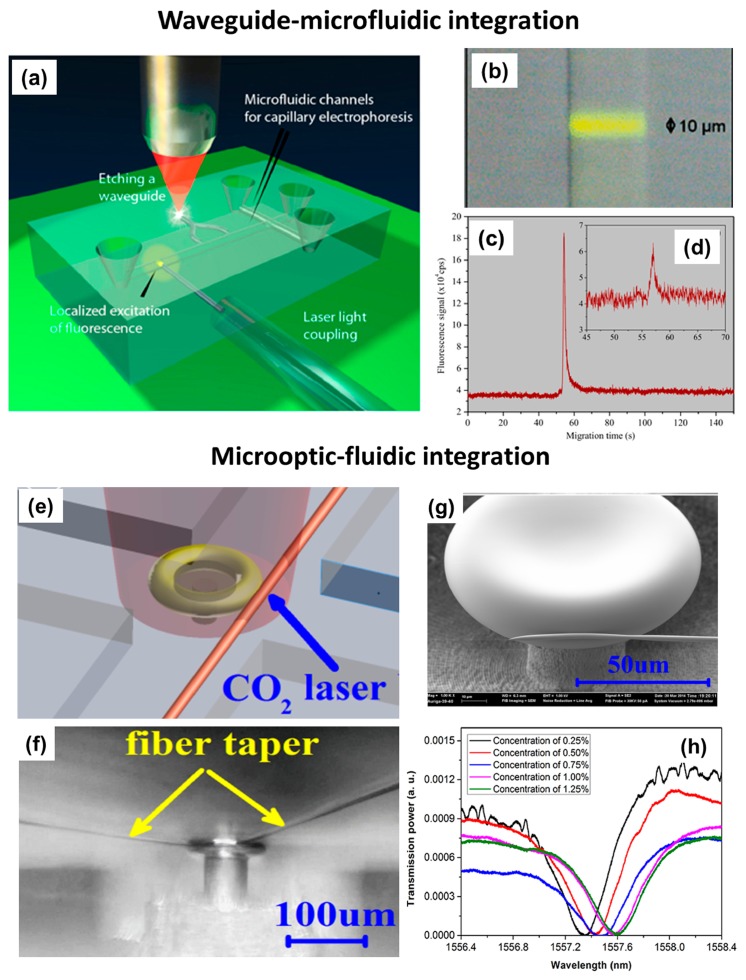
Waveguide-microfluidic integration and microoptic-fluidic integration using FsLDW, respectively. (**a**) Schematic illustration of FsLDW method used for adding optical waveguides to a lab-on-a-chip device for capillary electrophoresis; (**b**) Optical micrograph of the fluorescence signal excited in the microchannel by the optical waveguide. Electropherograms corresponding to (**c**) 10 nM and (**d**) 1 nM of dye-labeled oligonucleotides, measured at the end of the separation channel by the on-chip detection system [125] (Reproduced with permission from RSC. ^©^2009 by the Royal Society of Chemistry); (**e**) Schematic illustration of CO_2_ laser welding of a fiber taper to a microresonator fabricated by FsLDW; (**f**) Side-view optical micrograph of the fiber taper welded to the sidewall. The fiber remains adhered to the microtoroid even when it is bent. Note that the microtoroid is fabricated at the intersection of two open channels; (**g**) Close-up SEM image of the fiber taper welded to the sidewall of the microtoroid; (**h**) Optical absorption band at about 1557 nm for different salt concentrations [128] (Reproduced with permission from OSA. ^©^2014 by the Optical Society of America).

The FsLDW technique also allows optical waveguides to be fabricated in commercial microfluidic chips, in which a microchannel network has already been prepared and optimized [125]. In this way, an on-chip optical detection capability can be added without affecting the existing layout of the chip. Figure 12a schematically illustrates integration of optical waveguides into a commercial LOC for capillary electrophoresis. In this device, high-quality optical waveguides (diameter ∼10 μm) intersecting the separation channel were fabricated using FsLDW, and were used to detect biomolecules flowing through the channel with high spatial selectivity. To accommodate multipoint sensing, waveguide splitters can be fabricated with ease. Fluorescence from the optically excited volume was collected at a 90° angle by an optical fiber (not shown) glued to the bottom of the chip. To test the sensing capacity, the fluorescent dye Rhodamine 6G was used. As seen in the optical micrograph in Figure 12b, highly localized yellow fluorescence was observed in the waveguide when the channel was filled with a dye solution. The effectiveness of the device was validated by detecting different concentrations of dye-labeled oligonucleotides undergoing capillary electrophoresis on the chip. Figure 12c and d show electropherograms corresponding to concentrations of 1 and 10 nM of a Cy3-labelled 23-mer oligonucleotide, respectively, which illustrate the high sensitivity of the device.

Whispering-gallery-mode microcavities, which exhibit very high Q factors and small mode volumes, have recently been considered to be excellent choices for many high-efficiency sensing applications [126,127]. These components can also be fabricated using FsLDW. By integrating a microfluidic structure and an optical resonator, a microcavity-based sensor was reported [128]. Attachment of a fiber taper to the sidewall of a microtoroid was realized by welding using CO_2_ laser irradiation. As illustrated in Figure 12e, the fiber taper was first brought close to the microtoroid using a 3D nano-positioning stage to achieve critical coupling. The fiber taper was then moved slightly to bring it into contact with the sidewall of the microtoroid, and the microtoroid was irradiated with a CO_2_ laser beam. Figure 12f shows an optical micrograph of the microtoroid welded to the fiber taper, and Figure 12g shows a close-up SEM image of the welding point. The integrated microresonator has a high Q-factor of 3.21 × 10^5^ in air, which should be sufficient for many sensing applications. The functionality of the sensor was evaluated by measuring the bulk refractive index of purified water containing very small amounts of NaCl. Figure 12h shows optical absorption bands at a wavelength of about 1557 nm measured for different salt concentrations. Using this device, a detection limit of about 1.2 × 10^−4^ RIU (refractive index unit) could be achieved.

### 5.3. Electrofluidic Sensors

Microfluidic, microelectronic and optical technologies can be combined to develop LOC devices for highly reliable and sensitive biosensing and MEMS applications, thus hastening the development of electrofluidic and opto-electrofluidic devices. Such devices are highly attractive for next-generation high-throughput biochemical analysis and medical diagnostic systems, and have already been responsible for introducing Moore's law into the field of life sciences. Typical electrofluidic devices include electrowetting, electrophoretic and electrochromic devices. The interfacial tension between two immiscible fluids can be controlled using an electrical potential, and this basic principle is used in a diverse and continuously growing group of electro-optical modulated devices in which the optical state of liquids is changed by subjecting the liquid to an electric field or inducing electron transport. For example, it has been reported that using electrowetting, the meniscus between two immiscible liquids can act as a tunable optical lens for miniature cameras [129]. Electrophoresis is the motion of dispersed particles relative to a fluid under the influence of a spatially uniform electric field. It is ultimately caused by the presence of a charged interface between the particle and the surrounding fluid. It is the basis for a number of analytical techniques used in biochemistry for separating molecules by size, charge, or binding affinity. Electrochromism is a phenomenon displayed by some materials, in which a reversible color change occurs when a burst of charge is applied. It occurs due to electrochemical redox reactions that take place in electrochromic materials. Various types of materials and structures can be used to construct electrochromic devices, depending on the specific applications. For example, e-paper and e-ink are revolutionary display technologies based on electrochromism.

**Figure 13.**
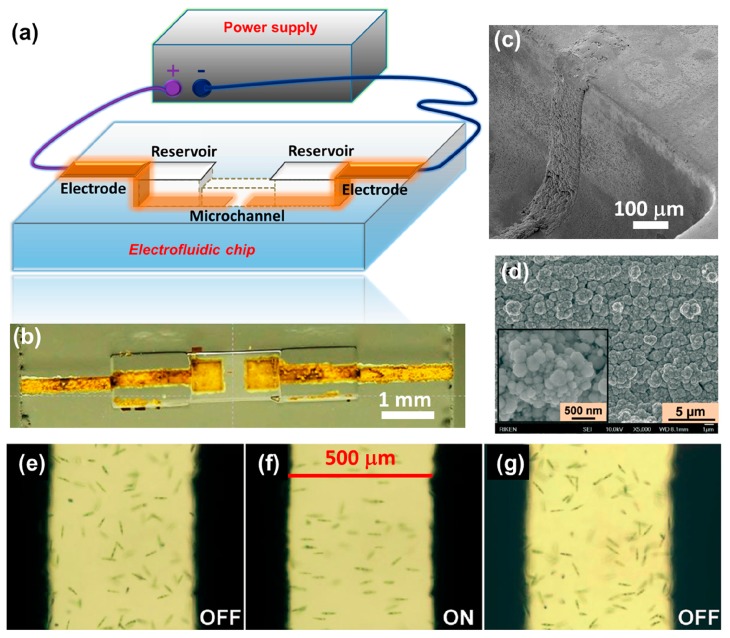
(**a**) Schematic of electrical wiring circuits formed in 3D microfluidic structure connected to an external power supply; (**b**) Photograph of electrofluidic device fabricated in Foturan glass chip using FsLDW (By courtesy of Jian Xu); (**c**) 45° tilted SEM image of metal structure on 350-μm-high sidewall after electroless copper plating; (**d**) SEM images of microstructure of deposited metal. The inset shows a higher-magnification image. Electro-orientation of *Euglena* cells in a microfluidic channel (**e**) before applying an electric field; (**f**) when an electric field (∼20 Vp-p, 0.9 MHz) was applied and (**g**) when the electric field was turned off. The electrode spacing in all images is 500 μm [99] (Reproduced with permission from RSC. ^©^2013 by the Royal Society of Chemistry).

Although no electrowetting or electrochromic based electrofluidic devices have so far been fabricated by FsLDW, they can easily be produced using the procedures described above. As a typical application of electrofluidic device fabrication, integration of electrical wiring circuits with 3D microfluidic structures [99] has been demonstrated, as shown in Figure 13. Such a device can be used to achieve electro-orientation of asymmetric living cells or microorganisms such as *Euglena gracilis* moving in a microfluidic channel. To fabricate this device, FsLDW followed by thermal treatment and chemical etching was first employed to produce a 3D microfluidic structure consisting of two reservoirs connected by a buried channel within a Foturan glass substrate. Taking advantage of the ablation caused by FsLDW, electroless metal plating was then carried out to produce patterned metal films forming a pair of microelectrodes around the reservoirs. The microelectrodes were connected to an external power supply, and an electric field was produced between the reservoirs, allowing electrofluidic manipulation of the cells. Figure 13a and b shows a schematic illustration and a photograph of this device, respectively. It should be noted that the key issue during the fabrication process is how to achieve metallization of the sidewalls of the microfluidic structure. To solve this problem, a volume writing method was developed that allowed uniform ablation of the sidewalls. The SEM images in Figure 13c and d demonstrate uniform metal structures embedded in the sidewall.

Microorganisms and cells can be oriented by applying an alternating current (AC) electric field, due to the interaction between the dipole moment induced by the electric field and the electric field itself. Figure 13e shows *Euglena* cells randomly swimming in the microchannel when no electric field was applied. In the presence of an appropriate electric field, the movement pattern changed significantly, with the cells rotating to orient themselves along the electrical field lines, as shown in Figure 13f. When the electric field was turned off, the initial random pattern was resumed, as shown in Figure 13g.

### 5.4. Surface-Enhanced Raman-Scattering Sensors

Since its discovery in the 1970s [130–132], SERS has provided a glimpse into the future of high-throughput single-molecule detection and analysis [133,134]. The high sensitivity of SERS spectroscopy and its inherent molecular specificity result in several distinct advantages over current state-of-the-art bioanalytical techniques, such as the ability to perform label-free analysis and to reduce photodamage effects. Much more information about samples can be obtained from Raman spectroscopy than from fluorescence spectroscopy, since all molecules have their own fingerprint Raman spectrum. Electrochemically reduced electrodes, colloidal clusters, and silver, gold or copper island films with nanoscale roughness can serve as SERS substrates.

The key issue with SERS lies in the fabrication of stable and reproducible substrates with a controllable enhancement factor (EF), which is by no means easy because of the difficulty in controlling the nanoscale behavior of materials. In order to achieve a high EF, a variety of fabrication techniques have been developed for SERS substrates, many of which consist of roughening the surface by some means and then depositing noble metal nanoparticles. Recently, FsLDW has been introduced as a powerful tool for fabricating SERS substrates [98,113,135,136].

It was first demonstrated by Zhou *et al.*, that it was possible to fabricate SERS substrates with a controllable EF using FsLDW on Ag-doped phosphate glass followed by electroless plating. In their investigation, silver seeds were first photoreduced using femtosecond laser irradiation, and were then transformed into silver nanoparticles with sizes suitable for SERS in the subsequent electroless plating process. Rhodamine 6G was then used as a probing molecule to investigate the enhancement of Raman signals from the substrate. The Raman signal was found to be enhanced almost uniformly over the entire substrate surface. In addition, using FsLDW, a microelectronic circuit and a micro-SERS unit were integrated into a microfluidic chamber, thus forming a prototype Raman chip for biosensing applications [113]. Furthermore, Lan *et al.* fabricated a SERS substrate on the tip of an optical fiber. FsLDW was first employed to etch uniform patterns on the end face of an optical fiber, which was then coated with a thin layer of silver by thermal evaporation [103].

**Figure 14.**
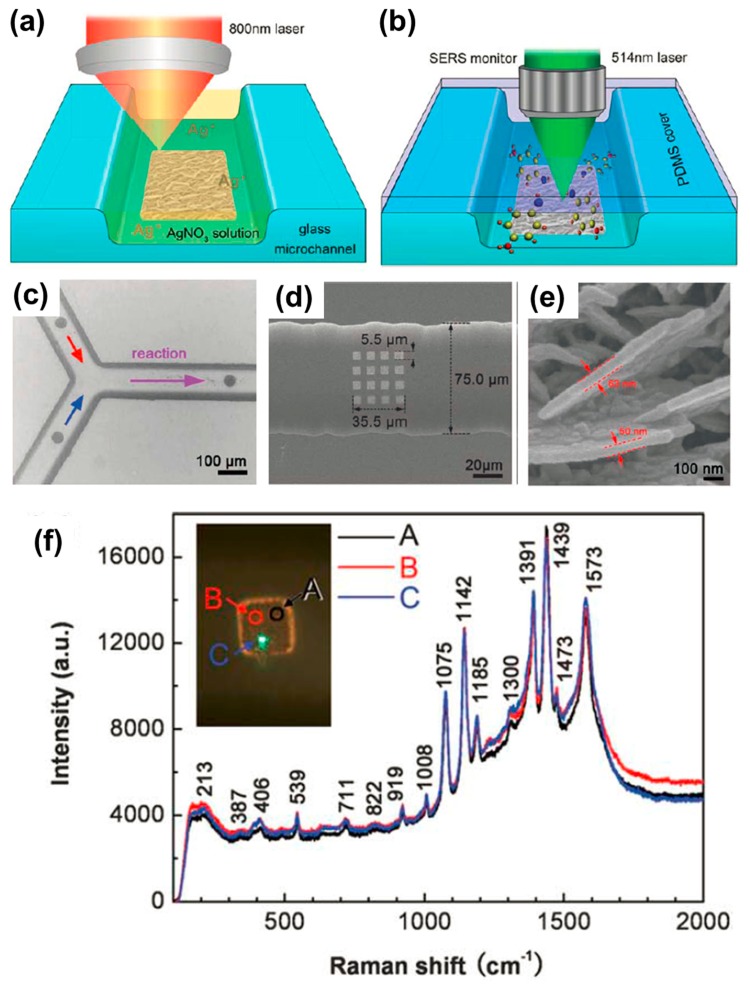
(**a**) Schematic illustration of fabrication process for silver SERS substrate inside a microfluidic channel; and (**b**) application for target molecule detection under visible light (514.5 nm) excitation; (**c**–**e**) SEM images of silver SERS substrates integrated into a microfluidic channel; (**f**) Raman spectra of p-aminothiophenol (*p*-ATP) measured at the positions shown in the optical micrograph in the inset [137] (Reproduced with permission from RSC. ^©^2011 by the Royal Society of Chemistry).

Recently, Xu *et al.* demonstrated the integration of high-efficiency SERS sensors into microfluidic channels [137]. FsLDW was adopted for highly localizable and controllable fabrication of SERS substrates by laser-induced photoreduction of a silver salt solution. The silver SERS substrates could be shaped into designed patterns, and could be precisely located at the desired position in the microchannel. Further investigation showed that the silver SERS substrates were composed of crystalline silver nanoplates with an average thickness of 50 nm. An enhancement factor of about 10^8^ was achieved for SERS detection when using *p*-aminothiophenol (*p*-ATP) as probing molecules, at an excitation wavelength of 514.5 nm. Figure 14 shows the fabrication method for a silver SERS substrate inside a microchannel and example applications. As seen in Figure 14a, a femtosecond laser pulse is tightly focused on the microchannel bed to fabricate the SERS substrate by photoreduction of the silver precursor. The microchannel is then sealed using a polydimethylsiloxane (PDMS) film for sensing applications under visible light excitation, as shown in Figure 14b. As a practical application example, Figure 14c shows three round silver SERS substrates fabricated at the branch of a Y-shaped microchannel. When two reactants pass the Y channel and mix together, this combination of substrates can detect both the reactants and the reaction products. Figure 14d shows a 4 × 4 array of 5.5 µm square silver SERS substrates, illustrating that a wide range of sensor configurations can be produced. In addition to fabricating such silver SERS substrates in glass, they can also be added to commercial PDMS chips. The surface morphology of the silver SERS substrate was characterized by SEM, and the results are shown in Figure 14e. It can be seen that nanoparticles are present on the surface of the nanoplates. This leads to increased roughness and a strong enhancement of the SERS signal. In a test using *p*-ATP, as shown in Figure 14f, the SERS signal was measured at three locations (A, B and C), and the difference in peak intensity was less than 3%, indicating the excellent uniformity of the SERS substrate.

### 5.5. Micro- and Nanofluidic Bioassays

In the fields of microbiology, cell biology and molecular biology, observations are usually performed using a glass slide with a coverslip, or using a Petri dish. However, the high numerical aperture objective lenses typically used for observations limit both the field of view and the depth of focus to several hundred micrometers, making it difficult and time-consuming to obtain clear images of rapidly moving microorganisms. Biologists urgently need to reduce observation times, not only to minimize the cost and time involved, but also due to limitations in the amount of computer memory available, which becomes problematic when acquiring movies using high-speed cameras. To this end, the use of microchips in which an entire room full of laboratory equipment can be shrunk and packed into a small space is being investigated [114]. In addition, in order to achieve high efficiency, accuracy and sensitivity, increasingly complex microchips are being fabricated in which microfluidic-based systems are integrated with electrical, mechanical, and optical components in a single chip. Femtosecond laser microfabrication is recently bringing these devices a step closer to reality, owning to it capacity for 3D integration of multiple functionalities. The ability to flexibly process a variety of glass allows fabrication of microfluidic sensors with feature sizes from millimeters to tens of nanometers, thus facilitating on-chip investigation of microorganisms, cells and even large biological molecules (e.g., protein and DNA molecules).

As a typical bioassay application, Hanada *et al.* proposed using microchips with 3D microfluidic structures to observe, analyze and manipulate microorganisms [114]. By using FsLDW in Foturan glass, two micro-reservoirs connected by a microfluidic channel were fabricated for accommodating aquatic organisms. With these microchips, the actual observation area can be restricted, but is still sufficient for the microorganisms to move freely, which makes it much easier to capture images of their movement. Furthermore, such microfluidic systems allow microorganisms to remain active for a long time, since little or no evaporation or leakage of water occurs. Such devices have been termed “nanoaquariums”. Using a combination of optical waveguides, filters and microfluidic structures formed by FsLDW, Hanada *et al.*, clarified the gliding mechanism of *Phormidium* [138]. It was found that CO_2_ secreted from seedling roots attracts *Phormidium* in the presence of light, and the light intensity and specific wavelength necessary for gliding were determined.

**Figure 15.**
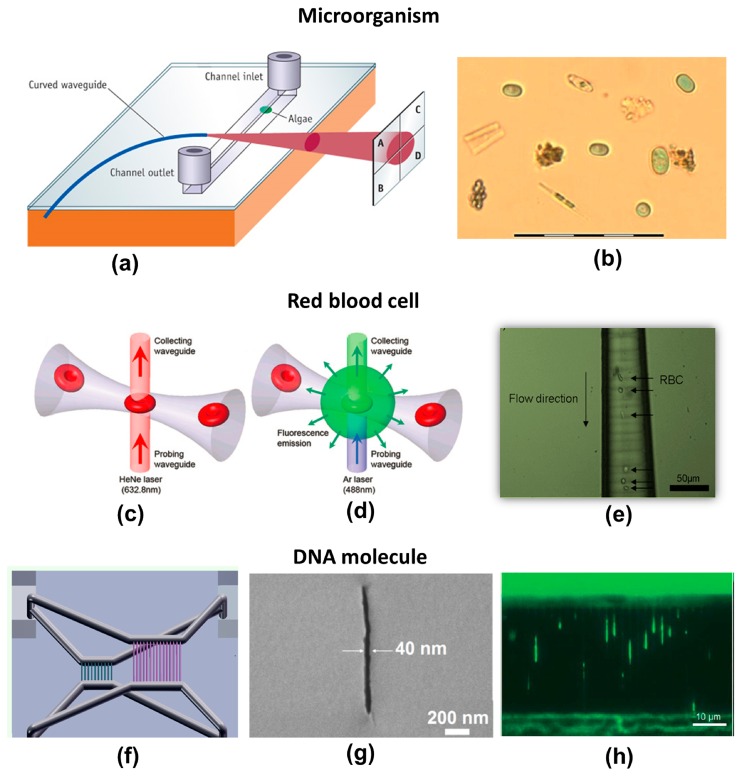
(**a**) Schematic illustration of a biosensor designed for fast screening, real-time monitoring, and initial classification of algae; (**b**) Micrograph of lab-cultured Cyanothece (green spheres/ellipses) amid detritus (other algae, plant matter) in field-collected sample. The scale bar represents 100 μm [139] (Reproduced with permission from RSC. ^©^2012 by the Royal Society of Chemistry). Schematic illustrations of cell detection using (**c**) transmittance and (**d**) fluorescence emission; (**e**) Red blood cells (RBCs) passing through a microchannel, forming a straight line in the flow [122] (Reproduced with permission from RSC. ^©^2009 by the Royal Society of Chemistry); (**f**) Schematic diagram of 3D nanofluidic device for DNA analysis; (**g**) Cross-sectional SEM micrograph of nanochannel fabricated in porous glass (**h**) Fluorescent images showing stretching of λ-DNA in nanochannels array with width of 50 nm [96] (Reproduced with permission from RSC. ^©^2013 by the Royal Society of Chemistry).

Schaap *et al.* demonstrated an example of a complete microsystem manufactured by FsLDW for optical classification of algae species [139]. As illustrated in Figure 15a, it consisted of a biochip designed for fast screening, real-time monitoring, and initial classification of algae. Rapid identification of algae species is a valuable tool for assessing water quality and monitoring adverse events such as eutrophication or phytoplankton “blooms” in response to an increase in nutrients such as nitrates or phosphates. The sensor incorporated both fluidic and optical functions. To fabricate the device, a square channel with a cross section of 100 μm × 100 μm was exposed and then etched in glass. A 90° curved waveguide was then formed perpendicular to the surface of the channel. The curvature of the waveguide eliminated uncoupled, parasitic light. The radius of curvature was 18 mm, determined by the laser-induced refractive index change. The sample-containing water flowed inside the fluidic channel. A fiber-coupled laser source was launched into the optical waveguide, illuminating a section of the channel. The transmitted light was then analyzed with a four-quadrant photodetector. If a cell or particle flows into a channel, it casts a shadow on the photodetector. The composite signal from the four quadrants is characteristic of the shape of the particle going through the channel. Figure 15b shows an optical micrograph of lab-cultured *Cyanothece* (green ellipsoids) amid detritus (other algae and plant matter) in a field-collected sample. It was experimentally demonstrated that nine different species of algae flowing into the same channel could be identified with an accuracy of 85%.

As another example of cell-biology applications, Figure 15c and d schematically illustrate a passive optical detection system used to measure the intensity of transmitted light and fluorescence emission, respectively [122]. The transmitted or fluorescence light was delivered by the collecting waveguide (top side) to the detector. The microfluidic channel in this system was first fabricated in fused silica using the technique described above. Longitudinal optical waveguides were then written by refractive index modification using FsLDW. The waveguides were normal to the top surface of the glass substrate so that they perpendicularly intersected the prefabricated microchannels. The diameter of the microchannels in the neck region where they intersected the optical waveguides could be varied between 5 and 100 µm by adjusting the etching time and the laser scanning parameters. The use of a neck diameter of 5 µm, which is slightly smaller than the size of a red blood cell (RBC) (6–8 µm, see Figure 15e), enabled a sharp, constant, and unambiguous signal for cell detection in both detection schemes. This is because healthy RBCs can squeeze through narrow microchannels with diameters down to 2 µm, so that all cells are detected.

Finally, buried nanofluidic channels with transverse widths down to less than 50 nm were directly created in porous glass substrates by combining the FsLDW threshold effect and formation of a periodic nanograting [20]. Using this technique, integrated micro-nanofluidic systems were produced by simultaneously writing micro- and nanofluidic channels with various 3D configurations, and were used for purposes such as investigating stretching of DNA molecules [96]. As shown in the schematic illustration in Figure 15f the device consists of two nanochannel arrays with different widths, lengths and depths embedded in a single glass substrate. A cross-sectional SEM micrograph of a nanochannel with a width of about 40 nm is shown in Figure 15g. The nanochannels are connected to four common reservoirs (2 mm × 2 mm × 500 µm depth) by 3D microchannels with diameters of about 50 µm. Such a device allows for simultaneous observations of stretching of DNA molecules in the two nanochannel arrays. Figure 15h shows a typical image of stretched λ-DNA in the nanochannels. In the thin nanochannels with a width of 50 nm, the average stretching length is 6.4 ± 1.0 µm (about 30% of the dye-adjusted contour length). Thus, this fabrication technique offers opportunities for developing novel 3D micro- and nanofluidic systems for a variety of LOC applications.

### 5.6. Femtosecond Laser Fabrication of Lab-on-Fiber Devices

Progress in the fabrication of advanced compact optical-fiber devices and the related development of manufacturing technologies has led to the “lab-on-fiber (LOF)” concept, which was first proposed by Cusanoa *et al.* [140,141]. A LOF system would integrate highly functional materials at the micro- or nanoscale within a single optical fiber, thus providing an “all-in-fiber” platform for both communications and sensing applications. LOF technology would thus represent the cornerstone of a photonics revolution enabling fiber-based multifunction sensor and actuator systems. It would offer unique advantages in terms of miniaturization, weight reduction, cost effectiveness, robustness, power consumption, and information control. Multifunctional laboratories integrated into a single optical fiber, exchanging information and combining sensory data, could provide effective automatic diagnostic features in addition to new photonic and electro-optic functionalities useful in fields such as optical processing, environmental management, life sciences, national security, and so on.

However, realization of such devices requires a reliable method for integrating structures and materials with different physical, mechanical, magnetic, chemical, and biological properties into an unconventional substrate such as the tip of an optical fiber, and forming the necessary interconnections. The currently proposed methods are mainly based on conventional approaches such as electron beam lithography and soft lithography [142]. Nevertheless, the optimum method for fabricating LOF devices is still hotly disputed.

**Figure 16.**
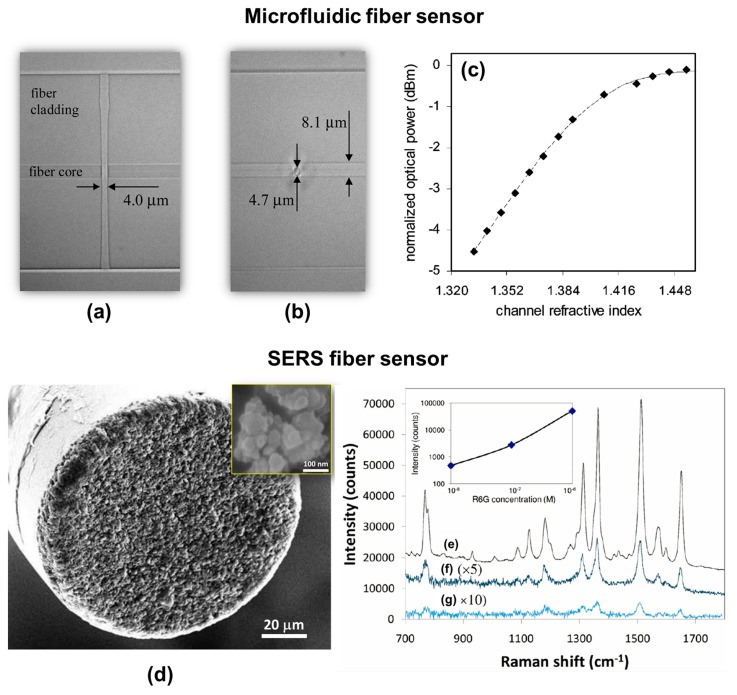
Microfluidic- and surface-enhanced Raman-scattering (SERS)-based fiber sensors fabricated by FsLDW, respectively. (**a**–**c**) Microfluidic channel fabricated in a single-mode fiber and (**d**–**g**) a SERS probe prepared on a multimode fiber tip for sensing applications. Optical micrographs of (**a**) top-view and (**b**) cross-sectional view of the microchannel within the fiber; (**c**) Normalized optical power transmitted through the microfluidic fiber device for different microchannel refractive indices [143] (Reproduced with permission from OSA. ^©^2006 by the Optical Society of America); (**d**) SEM image of femtosecond laser-ablated fiber end-face. The inset shows an SEM image of silver nanoparticles coated on the structured surface. Raman spectra of R6G solutions with different concentrations obtained using a 1-m-long fiber SERS probe: (**e**) 10^−6^ M; (**f**) 10^−7^ M (multiplied by 5 for easy viewing); (**g**) 10^−8^ M (multiplied by 10 for easy viewing). The inset shows the dependence of the SERS intensity at 1511 cm^−1^ on the R6G concentration [103] (Reproduced with permission from OSA. ^©^2009 by the Optical Society of America).

Even before the LOF concept was proposed in the 2011, the rapid development of femtosecond laser microfabrication techniques led to the idea of fiber-based sensing devices, which were also referred to as “all-in-fiber” devices. In 2006, for the first time, Lai *et al.* demonstrated a microfluidic fiber device for refractive index sensing, consisting of a 4-µm-wide microchannel intersecting the fiber core, which was fabricated by femtosecond laser processing and chemical etching [143]. The device structure is shown in Figure 16a and b. To demonstrate its sensing capabilities, different volume concentrations of glycerine in water were prepared to create solutions with refractive indices ranging from 1.333 to 1.475. Figure 16c shows the dependence of the normalized power at 1538 nm transmitted through the microfluidic fiber device on the refractive index of the liquid in the channel. Since this initial report, there have been a range of studies on the formation of holes and channels in optical fibers either by direct femtosecond laser ablation or assisted by wet chemical etching, allowing measurement of properties such as the refractive index, temperature, and vibration characteristics, even in harsh environments [144–150]. The operating principle for most of these devices was based on interferometry, with structures such as a Fabry–Perot cavity or an MZI.

Taking advantage of the ease of producing FBG structures using FsLDW, long-period fiber gratings and even fiber lasers were demonstrated [69,151–154] for remote sensing applications, and these can also be considered as all-in-fiber devices. Using selective metallization, SERS probes were prepared on fiber tips using FsLDW [103,136], as shown in Figure 16d. Femtosecond laser ablation was used to produce nanostructures on the cleaved end-face of a multimode optical fiber with core and cladding diameters of 105 and 125 µm, respectively. Laser ablation can create a roughened surface with quasi-uniformly distributed features with sizes of several hundred nanometers. The fiber end-face was then SERS-activated by silver electroless plating. In SEM images, the coated silver took the form of isolated nanoparticles with sizes ranging from 20 to 60 nm, as shown in the inset in Figure 16d. Figure 16e plots Raman spectra of R6G solutions with various concentrations (10^−6^–10^−8^ M) obtained using the fiber SERS probe. With a laser excitation power of 1.7 mW and an integration time of 1 s, the lowest detectable R6G concentration was about 10^−8^ M. The Raman intensity at 1511 cm^−1^ is plotted as a function of R6G concentration in the inset of Figure 16e for reference.

**Figure 17.**
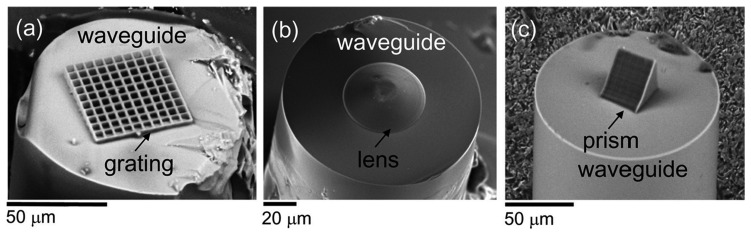
SEM images of (**a**) tilted micrograting; (**b**) microlens; and (**c**) microprism formed by photopolymerization on an optical fiber tip using FsLDW [101] (Reproduced with permission from IOP. ^©^2012 by the Institute of Physics).

Using additive TPP processing, Malinauskas *et al.*, reported the integration of fine 3D micro-optical components on a fiber tip [101]. Although the effectiveness and versatility of laser polymerization has already been established by fabricating single optical elements with complex surface shapes, such as aspherical, Fresnel, and solid immersion lenses [155,156], this additive TPP processing enables multiple optical functions and hybrid refractive-diffractive optical elements to be integrated into conventional devices such as optical fibers. A diffractive grating, a lens, and a prism have been successfully integrated into the tip of an optical fiber (see Figure 17). This allows the introduction of new functionalities in a variety of sensing applications in fields such as optomechanics and optofluidics. Although the above LOF devices have only a single function, they can act as important building blocks for future LOF or all-in-fiber systems.

## 6. Conclusions and Outlook

Femtosecond laser microfabrication in glass has become a powerful technique for producing microfluidic sensors. In particular, it allows the incorporation of different components into a monolithic chip to create new exciting functionalities. Compared with existing techniques, this approach is based on a unique MPA process within an ultrashort duration, which offers the advantages of eliminating thermal diffusion, internal processing, sub-diffraction-limited resolution and the capability of multifunctional integration. It has been successfully used to fabricate a wide variety of integrated microfluidic sensors, such as complex 3D micro- and nanofluidic networks, electrofluidic and optofluidic sensors, SERS chips and LOF devices.

Several issues need to be addressed in the future. First, to meet the requirements of low-loss optics and smooth fluidics applications, improved methods are needed for smoothing the inner surfaces of devices fabricated in porous glass substrates. The existing approaches such as annealing, oxygen/hydrogen flame polishing, and CO_2_ laser reflow has enabled creation of smooth surfaces of optical lenses and microresonators and smooth inner walls of microfluidic channels of relatively large diameters (*i.e.*, diameters of tens of microns), they become increasingly less effective when the size of microfluidic structure decreases. In addition, although a feature size of λ/20 has been achieved in porous glass in the transverse direction, it is still challenging to create nanofluidic channels of fully nanometer-scale dimensions in glass substrates. Selective metallization using FsLDW is expected to be applied to a variety of sensing applications such as electrofluidics, SERS sensors, and plasmonics. However, although sidewall metallization has been successfully demonstrated, fabrication of 3D freestanding metal structures in closed microfluidic channels has not. Finally, multifunctional integration is still not easy to achieve for a LOF or an all-in-fiber device due to the special shape of the fiber (*i.e.*, cylindrical sidewall). It is expect that these difficulties will be gradually overcome in the future. Recently, a new ship-in-a-bottle integration strategy has been proposed, in which 3D functional microcomponents are created inside pre-fabricated 3D microfluidic structures [102]. Such a unique approach is only possible by FsLDW, and is highly promising for flexible fabrication of highly functional microfluidic sensors.

As a final note, efforts should be made to bridge the gap between current fundamental research and future commercial applications of microfluidic sensors fabricated by FsLDW. Thanks to the development of stable, reliable femtosecond laser systems with high output power, femtosecond laser processing has been already used for some industrial and commercial applications in the automotive, electronics, and medical fields. For commercialization of disposable microfluidic sensors, however, further reductions in fabrication cost are required, in addition to improving the throughput.

## Figures and Tables

**Table 1.** Typical properties and applications of fused silica, Foturan glass and porous glass.

**Glass**	**Properties Related to FsLDW**	**Applications**
Fused silica	Composition: amorphous SiO_2_ Refractive index: 1.46 (550 nm) Melting temperature: 1665 °C Annealing temperature (for surface smoothing): 1200 °C [89] High optical transparency, low background fluorescence, biocompatible. Selective etching ratio: ∼1:30 (HF), ∼1:200 (KOH) [85]	Photonic devices [38–43] Microfluidics [82,83,85,87,107] Optofluidic integration [25,45,108,109] Micro-optics [88–90,110] Micromechanics [111,112] Microelectronics [97,113] Surface/bulk nanostructuring [48,49]
Foturan	Composition: lithium-potassium glass dotted with small amounts of silver and cerium oxides Refractive index: 1.52 (550 nm) Annealing temperature (for surface smoothing): 570 °C Melting temperature: 660 °C Selective etching ratio: ∼1:50 (HF)	Microfluidics [91,114] Micro-optics [115,116] Optofluidic integration [25,45–47,117] Micromechanics [118] Microelectrodes [119] Electrofluidics [99]
Porous glass	Composition: 95.5SiO_2_-4B_2_O_3_-0.5Na_2_O (wt.%). * Mean pore size: ∼10 nm * Relative pore volume: 40% Annealing temperature (for sealing): 1120 °C Microfluidic channels with arbitrary 3D geometries and unlimited length Feature size approaching ∼λ/20	Micro and nanofluidics [94–96] Bulk nanostructuring [96]

* The mean pore size and relative pore volume for the porous glass are taken from Refs. [94–96]. In fact, these parameters can be tuned by adjusting the preparation conditions for the glass.
